# Increasing survivors of anthracycline-related cardiomyopathy with breast cancer in trastuzumab era: thirty-one-year trends in a Japanese Community

**DOI:** 10.1007/s12282-024-01623-0

**Published:** 2024-08-13

**Authors:** Mitsuhiro Watanabe, Shinya Fujiki, Yuji Okura, Chie Toshikawa, Mayuko Ikarashi, Chizuko Kanbayashi, Koji Kaneko, Akira Kikuchi, Eiko Sakata, Keiichi Tsuchida, Kazuyuki Ozaki, Kazuki Moro, Naoki Kubota, Takeshi Kashimura, Masato Moriyama, Nobuaki Sato, Naohito Tanabe, Yu Koyama, Toshifumi Wakai, Yasuo Saijo, Takayuki Inomata

**Affiliations:** 1 Department of Cardiovascular Medicine, Niigata University Graduate School of Medical and Dental Sciences, Niigata, Japan; 2 Department of Onco-Cardiology, Niigata Cancer Center Hospital, 2-15-3 Kawagishi-cho, Chuo-ku, Niigata, 951-8560 Japan; 3 Division of Digestive and General Surgery, Niigata University Graduate School of Medical and Dental Sciences, Niigata, Japan; 4 Department of Breast Surgery, Niigata City General Hospital, Niigata, Japan; 5 Department of Breast Oncology, Niigata Cancer Center Hospital, Niigata, Japan; 6 Department of Gynecology, Niigata Cancer Center Hospital, Niigata, Japan; 7 Department of Cardiology, Niigata City General Hospital, Niigata, Japan; 8 Department of Pathophysiology, Faculty of Pharmacy, Niigata University of Pharmacy and Medical and Life Sciences, Niigata, Japan; 9 Department of Health and Nutrition, Faculty of Human Life Studies, University of Niigata Prefecture, Niigata, Japan; 10 Department of Nursing, Niigata University Graduate School of Health Sciences, Niigata, Japan; 11 Department of Medical Oncology, Niigata University Graduate School of Medical and Dental Sciences, Niigata, Japan

**Keywords:** Cardiotoxicity, Anthracycline, Trastuzumab, Human epidermal growth factor receptor type 2, Secular trend

## Abstract

**Background:**

Trastuzumab has improved breast cancer (BC) prognosis and reduced anthracycline use. However, the characteristic changes of anthracycline-related cardiomyopathy (ARCM) in patients with BC remain unclear. We aimed to update our understanding of ARCM in the trastuzumab era.

**METHODS:**

This retrospective observational cohort study included 2959 patients with BC treated with anthracyclines at three regional cancer centers in Niigata City between 1990 and 2020. Seventy-five patients (2.5%) developed ARCM and were categorized into two groups: pre- 2007 (early phase) and post-2007 (late phase), corresponding to before and during the trastuzumab era in Japan.

**Results:**

ARCM incidence peaked at 6% in the 1990s, then decreased and stabilized at 2% until the 2010s. Survivors of anthracycline-treated BC increased more rapidly in the late phase, with four times as many patients with ARCM compared to the end of the early phase (26 and six, respectively). Although the rate of change in accumulation from the early phase to the late phase was slight in the anthracycline-treated BC group, it was more pronounced in the ARCM group (*P* < 0.001). Mean anthracycline use in the late phase was significantly lower than in the early phase (307 vs. 525 mg/m^2^, *P* < 0.001). Five-year survival rates in the late phase tended to be higher than early phase (45% and 28%, respectively. *P* = 0.058). Human epidermal growth factor receptor type 2 (HER2) positivity with trastuzumab therapy in the late phase was an independent predictor for mortality within 10 years (hazard ratio = 0.24, 95% confidence interval: 0.10–0.56; *P* = 0.001).

**Conclusions:**

HER2-positive patients with ARCM receiving trastuzumab therapy had a better prognosis than HER2-positive and HER2-negative patients with ARCM not receiving trastuzumab therapy, and this trend has been increasing in the trastuzumab era. These findings highlight the importance of HER2-targeted treatments in improving prognosis for BC patients with ARCM.

**Supplementary Information:**

The online version contains supplementary material available at 10.1007/s12282-024-01623-0.

## Introduction

Breast cancer (BC) is the most prevalent malignancy in Western countries, and its worldwide prevalence is expected to increase significantly in the future [1–3]. In East Asia, the incidence of BC has increased rapidly over the past few decades, making it the most common cancer in several countries, including Japan [4, 5]. Although its prognosis has improved [6], a significant risk of cancer treatment-related cardiac dysfunction (CTRCD) persists, which has been associated with potential treatment termination and lower therapeutic efficacy [7–12]. Therefore, cancer treatment-related cardiotoxicity is of significant concern.

In patients with BC, anthracycline or trastuzumab is the most common cause of CTRCD. Anthracyclines induce myocardial damage and necrosis in a dose-dependent manner through oxidative stress and other mechanisms. Cardiomyocytes, being unable to repair or regenerate, undergo irreversible injury, leading to cardiotoxicity [7–10]. In contrast, trastuzumab, an anti-human epidermal growth factor receptor type 2 (HER2) antibody, can cause cardiomyocyte dysfunction, but not necrosis, and its induced cardiotoxicity is reversible [8–12]. CTRCD caused by these treatments increases the risk of death and symptomatic heart failure (HF) in the future. Considering the remarkably younger age at diagnosis in patients with BC compared to those with other malignancies, its social impact, defined by mortality and living with a disability, is also prominent.

Although anthracycline doses have historically been restricted to avoid cardiotoxicity [9, 10, 13, 14], most patients receive anthracycline-containing chemotherapy as neoadjuvant or adjuvant therapy [12–15]. Patients with recurrent/metastatic BC have been forced to receive anthracycline up to the limits of its use [16]. In contrast, trastuzumab has shown high efficacy in patients with HER2-positive BC since its introduction in the early 2000s. Trastuzumab partially replaced traditional anthracycline-containing regimens in both early and advanced cancer and reduced anthracycline use by preventing recurrence/metastasis in the mid-2000s [6, 10, 12, 14, 16]. Consequently, trastuzumab may have changed not only the cancer prognosis but also anthracycline-related cardiomyopathy (ARCM). However, the impact of this therapeutic revolution on ARCM remains unknown, particularly in the Asian community. To update our knowledge of ARCM in the trastuzumab era, we evaluated patients with BC and ARCM over the past 31 years in a local Japanese city.

## Methods

### Design and study cohort

This retrospective observational multicenter cohort study, based on clinical data, was performed in three designated cancer care hospitals in Niigata City (Niigata University Medical and Dental Hospital, Niigata Cancer Center Hospital, and Niigata City General Hospital). Hospital-based BC registries in Japan, designated for cancer care hospitals nationwide since 2004, collect information on therapies and prognoses for all cancer cases encountered in each hospital according to coding rules precisely defined by the Japanese Breast Cancer Society [17, 18]. These three local hospitals designated for cancer care have registered the medical information of 97% of the patients with BC in Niigata City. Data before 2004 were comprehensively collected from the information databases for cancer in each hospital.

Consecutive patients diagnosed with BC and treated with two or more anthracycline treatments between January 1, 1990, and December 31, 2020, were identified. We investigated all echocardiography examinations performed after anthracycline treatment and defined ARCM as patients newly diagnosed with a left ventricular ejection fraction (LVEF) < 50% [19, 20]. Patients with LVEF < 50% before BC therapy were excluded.

To assess the impact of the recommended therapeutic strategies in the clinical practice guidelines, we divided the patients into two groups based on the phases of study enrollment: before 2007 (early phase) and after 2007 (late phase). Since 2007, Japanese BC clinical practice guidelines have recommended trastuzumab for HER2-positive patients in both early and advanced settings [21]. Therefore, the early and late phases were equivalent to before and in the trastuzumab era in Japan, respectively.

### Data collection

Data on the baseline patient characteristics and treatments associated with BC and cardiomyopathy were collected. The cumulative anthracycline dose was calculated by converting the different anthracycline doses into doxorubicin equivalents [22]. The incidence of ARCM was calculated by dividing the number of patients with ARCM by the total number of patients treated with anthracyclines.

### Ethical considerations

This study was approved by the institutional review board and independent ethics committee of each participating site. A waver of informed consent was approved. It was conducted in accordance with the principles of the Declaration of Helsinki and in compliance with the ethical guidelines for medical and health research involving human participants. This study was registered with the University Hospital Medical Information Network of Japan (registration number UMIN-000052011).

### Statistical analysis

To calculate the significance of differences between the two groups, Student’s t-test was used for continuous variables, and Fisher's exact probability test was used for categorical variables. Values are reported as mean ± standard deviation and numbers (%). Non-parametric Mann–Whitney’s *U* test was used to compare differences between median months from the last dose of anthracycline to ARCM. Linear regression analysis was used to examine the effect of de novo patient accumulation on the number of surviving patients.

Survival curves and cumulative incidence rate curves were evaluated using the Kaplan–Meier method, and differences between groups were assessed using a log-rank test. Factors associated with death within 10 years were analyzed using Cox proportional hazards models. For the multivariable models, independent variables were selected from the variables that were significant in the crude models. IBM SPSS Statistics for Windows, version 27.0; (Armonk, NY, IBM Corp) was used for the statistical analyses, and the significance level was set at a two-tailed *P* < 0.05.

## Results

### Number of patients treated with anthracyclines and patients diagnosed with ARCM

**Fig. 1:**
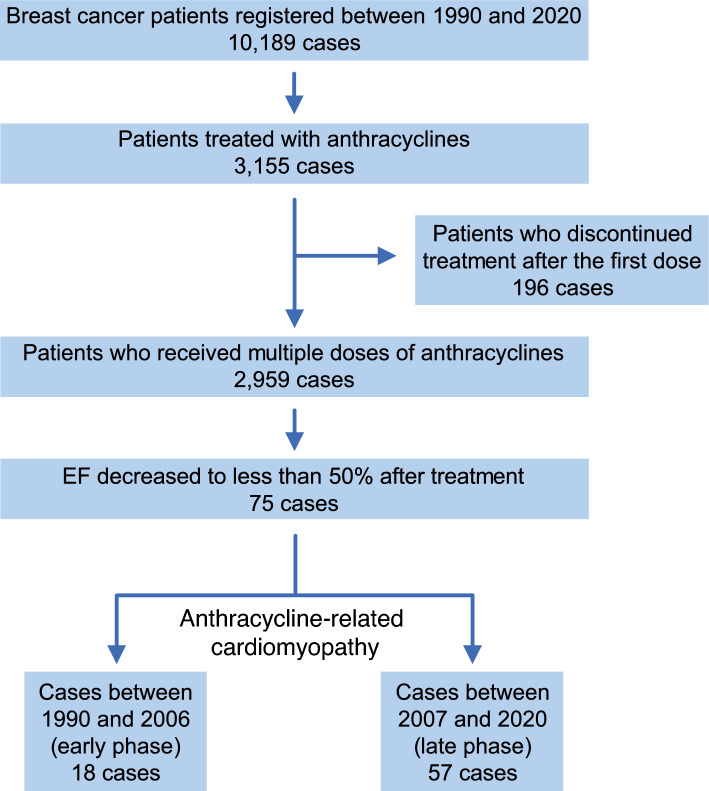
Patient flowchart for the study

**Fig. 2:**
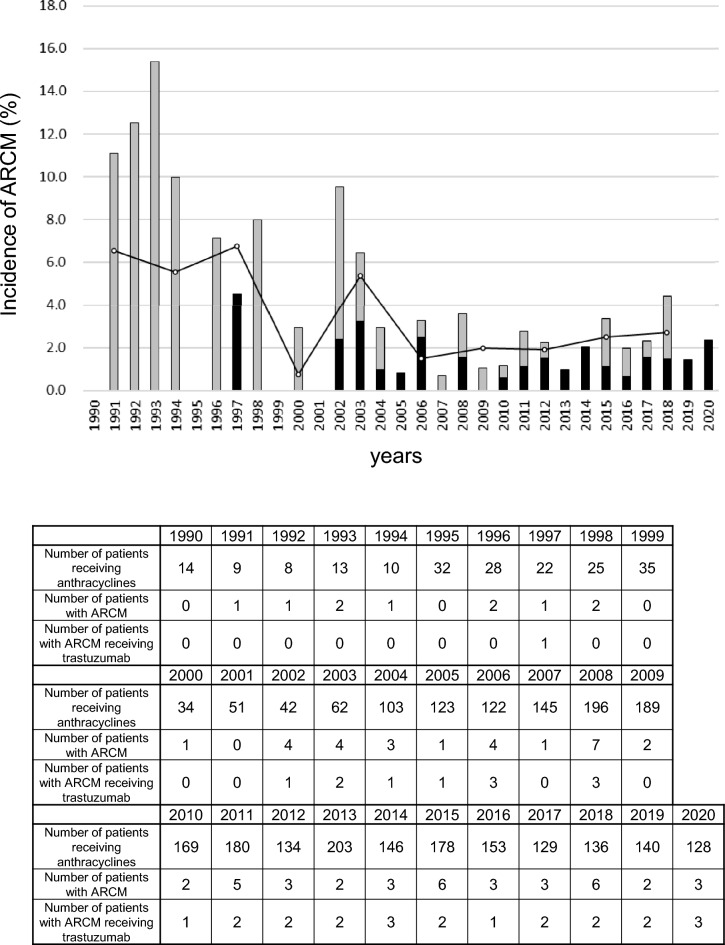
Incidence of ARCM in anthracycline-treated patients. The incidence was calculated by dividing the number of patients who developed ARCM later by the number of patients who started anthracyclines each year. Black bars indicate patients treated with trastuzumab; grey bars indicate patients not treated with trastuzumab. The line graph shows the incidence of ARCM every 3 years

A total of 10, 189 women were diagnosed with BC between January 1990 and December 2020. Of these, 2,959 patients were treated at least twice with anthracyclines. The median follow-up from the start of anthracycline administration was 6.3 [interquartile range (IQR) 3.1–10.2] years. Consequently, 75 patients (2.5%) were diagnosed with ARCM (Fig. 1). The incidence of ARCM calculated every 3 years was approximately 6% in the 1990s but subsequently decreased to approximately 2% in the 2010s (Fig. 2). The cumulative incidence of ARCM in all patients was 2.1% over 5 years and 3.2% over 10 years (Fig. S1a). In the early phase, the incidence was 3.0% over 5 years and 4.7% over 10 years, whereas in the late phase, it was 1.8% and 2.7%, respectively (P = 0.047) (Fig. S1b).

**Fig. 3:**
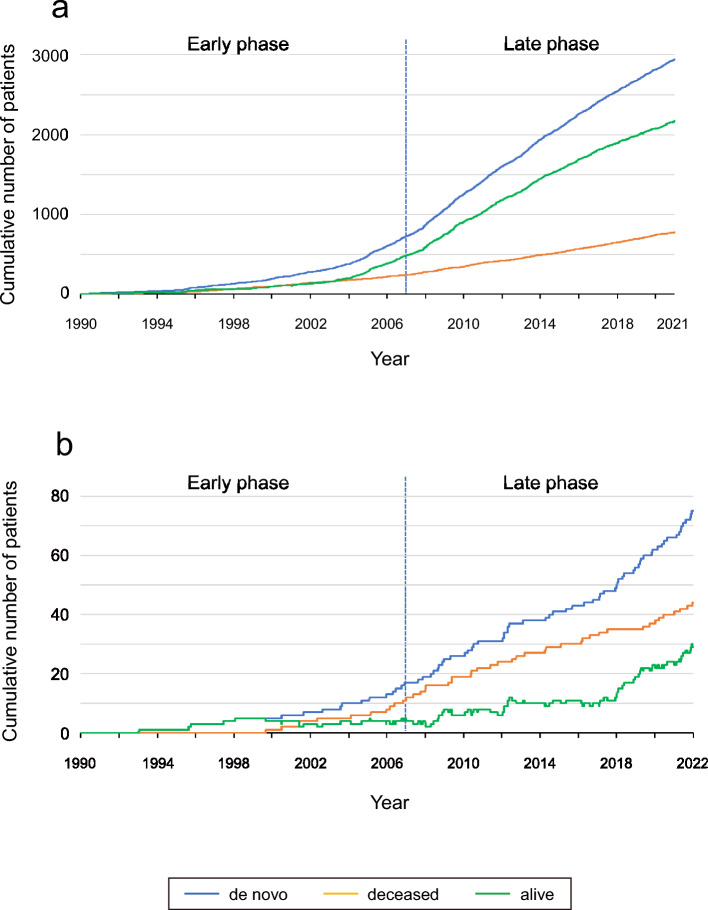
Cumulative number of BC patients treated with anthracycline and ARCM. **a** Cumulative number of BC patients treated with anthracycline from January 1, 1990, to December 31, 2020. The number of patients was counted based on the date of the first anthracycline administration. **b** Cumulative number of patients with ARCM from January 1, 1990, to December 31, 2021. The number of patients was counted based on the date of diagnosis of ARCM. The blue line represents de novo cancer patients, the green line represents living patients, and the orange line represents deceased patients

Among patients treated with anthracyclines, the cumulative number of survivors outnumbered the cumulative number of deaths since 2005 (Fig. 3a). In contrast, among patients with ARCM, the cumulative number of survivors was lower than that of patients who died (Fig. 3b). However, a rapid increase in the number of ARCM survivors was observed during the late phase. To evaluate whether this increase in the accumulation speed was affected solely by the increase in that of de novo patients, the regression coefficients of de novo patients predicting that of alive patients were compared between the two periods. In the anthracycline-treated patient group, the regression coefficients were significantly, but only slightly, elevated in the late phase than in the early phase (+ 0.010, *P* < 0.001) (Table S1 and Fig. S2a). However, the increase in the coefficients was much greater in the ARCM group than in the anthracycline-treated patient group (+ 0.110, *P* < 0.001) (Fig. S2b).

### Characteristics of patients with ARCM

The characteristics of the 75 patients with ARCM are presented in Table 1. Fifty-seven percent of the patients had distant metastases, and 52% had HER2-positivity. Doxorubicin and epirubicin were the main drugs used for chemotherapy, with a mean doxorubicin-equivalent dose of 359 mg/m^2^. In the early phase, only 17% of patients received trastuzumab, but in the late phase, 54% of patients received it. All but one of these trastuzumab-treated patients were HER2 positive. The average number of echocardiography performed before diagnosing ARCM was low, with 0.7 ± 0.5 in the early phase and 2.0 ± 2.0 in the late phase (P = 0.017). The median duration from the last treatment to the diagnosis of ARCM was 14.3 months (IQR, 4.6–33.9), LVEF at that time was 41.9 ± 7.8%, and LVEF of 16 patients (21%) was < 35%. Twenty-nine patients (39%) required hospitalization for HF after ARCM diagnosis. In the late-phase group, patients had fewer metastases, used more taxanes and trastuzumab, and were administered fewer anthracyclines than patients in the early-phase group; furthermore, the late-phase group had more patients with HER2-positive tumors. Examination of plasma brain natriuretic peptide (BNP) levels and HF treatment with angiotensin-converting enzyme inhibitors (ACEIs) and beta blockers were more common in the late phase. No differences were observed in the duration until LVEF declined after anthracycline treatment, LVEF levels, or the frequency of hospitalization for congestive HF.

**Table 1:** Patient characteristics

	Total (*N* = 75)	Early phase (*N* = 18)	Late phase (*N* = 57)	*P* value
Age at ARCM diagnosis, years	59.3 ± 8.9	58.4 ± 9.2	59.6 ± 8.9	0.62
Height, cm	156.1 ± 6.0	153.8 ± 5.9	156.8 ± 5.8	0.06
Body weight, kg	55.5 ± 9.3	57.2 ± 10.5	55.0 ± 8.9	0.39
Body mass index, kg/m^2^	22.8 ± 3.4	24.1 ± 3.8	22.3 ± 3.2	0.06
Total protein, g/dL	6.7 ± 0.6	6.3 ± 0.7	6.8 ± 0.6	0.012
Total cholesterol, mg/dL	191.4 ± 52.0	175.8 ± 59.7	196.1 ± 49.0	0.16
Serum creatinine, mg/dL	0.7 ± 0.5	0.6 ± 0.1	0.7 ± 0.5	0.27
Hemoglobin, g/dL	11.8 ± 1.8	11.4 ± 1.6	11.9 ± 1.8	0.37
NT-proBNP, pg/mL^a^			2014 ± 2917	
Ever smoker, *n* (%)	8 (10.7)	0 (0)	8 (14.0)	0.19
Hypertension, *n* (%)	13 (17.3)	2 (11.1)	11 (19.3)	0.72
Diabetes mellitus, *n* (%)	6 (8.0)	3 (16.7)	3 (5.3)	0.15
Dyslipidemia, *n* (%)	7 (9.3)	0 (0)	7 (12.3)	0.19
Coronary artery disease, *n* (%)	3 (4.0)	0 (0)	3 (5.3)	1.00
Hormone receptor				
Estrogen receptor, *n* (%)	42 (56.8)	9 (50.0)	33 (58.9)	0.59
Progesterone receptor, *n* (%)	32 (42.7)	7 (38.9)	25 (43.9)	0.79
HER2 positivity, *n* (%)^b^	35 (52.2)	3 (27.3)	32 (57.1)	0.10
Triple negative	16 (21.3)	7 (38.9)	9 (15.8)	0.050
Metastasis, *n* (%)	43 (57.3)	17 (94.4)	26 (45.6)	< 0.001
Surgery, *n* (%)	70 (93.3)	16 (88.9)	54 (94.7)	0.59
Radiotherapy, *n* (%)	46 (61.3)	11 (61.1)	35 (61.4)	1.00
Left chest radiation, *n* (%)	17 (22.7)	2 (11.1)	15 (26.3)	0.22
Chemotherapy agents				
Doxorubicin, *n* (%)	40 (53.3)	13 (72.2)	27 (47.4)	0.10
Epirubicin, *n* (%)	37 (49.3)	7 (38.9)	30 (52.6)	0.42
Taxane, *n* (%)	56 (74.7)	9 (50.0)	47 (82.5)	0.011
Trastuzumab, *n* (%)	34 (45.3)	3 (16.7)	31 (54.4)^d^	0.006
Doxorubicin equivalent cumulative dose, mg/m^2^	359 ± 198.8	525 ± 133.1	307 ± 187.6	< 0.001
Median months from the last dose to ARCM (interquartile range)	14.3 (4.6–33.9)	10.7 (0.6–22.3)	14.3 (6.1–46.4)	0.11
LVEF at ARCM diagnosis^e^, %	41.9 ± 7.8	43.8 ± 5.9	41.3 ± 8.2	0.23
Hospitalized HF, *n* (%)	29 (38.7)	8 (44.4)	21 (36.8)	0.59
Medications for HF^f^, *n* (%)	41 (54.7)	7 (38.9)	34 (59.6)	0.12
ACEIs or ARBs	32 (42.7)	3 (16.7)	29 (50.9)	0.014
Beta blockers	28 (37.3)	3 (16.7)	25 (43.9)	0.051
MRAs	28 (37.3)	6 (33.3)	22 (38.6)	0.78

Data are mean ± SD or *n* (%)

*ACEIs* angiotensin-converting-enzyme inhibitors, *ARBs* angiotensin receptor blockers, *ARCM* anthracycline-related cardiomyopathy, *HER2* human epidermal growth factor receptor type2, *HF* heart failure, *LVEF* left ventricular ejection fraction, *MRAs* mineralocorticoid receptor antagonists, *NT-proBNP* N-terminal pro-brain natriuretic peptide

^a^NT-proBNP was hardly measured in early phase, so it is not presented

^b^HER2 was not evaluated in seven cases in early phase, and one case in late phase, respectively because HER2 testing was not available. HER2-positive status refers to the strong HER2 protein expression or amplification of the HER2/neu gene, which is determined by either: an immunohistochemistry score of 3 + or a positive fluorescence in situ hybridization result

^c^Five cases were double negative in the absence of HER2 testing

^d^One out of 31 treated cases was HER2 negative: HER2 positivity with trastuzumab therapy was 3 (16.7%) in the early phase and 30 (52.6%) in the late phase (P = 0.013)

^e^Three cases (16.7%) in the early phase and 13 (22.8%) in the late phase was less than 35%

^f^Patients treated with ACEI, ARBs, beta blockers, or MRAs

### Differences in prognosis between the phases of patient enrollment

**Fig. 4:**
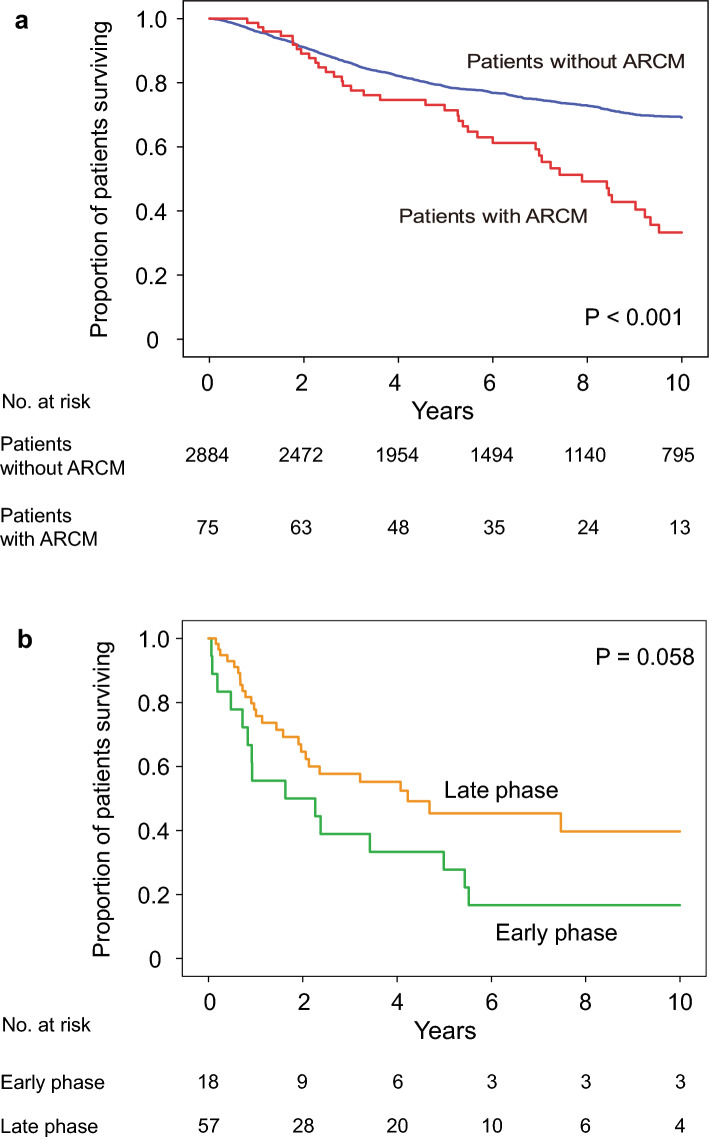
Kaplan–Meier estimates of overall survival. **a** Overall survival in patients with BC with or without ARCM from the first day of anthracycline administration. The blue line represents patients without ARCM and the red line represents patients with ARCM. Statistically significant differences were determined using the log-rank test. **b** Overall survival in patients with BC and ARCM from the diagnosis of ARCM according to two phases. The early and late phases are indicated by the green and orange lines, respectively

**Fig. 5:**
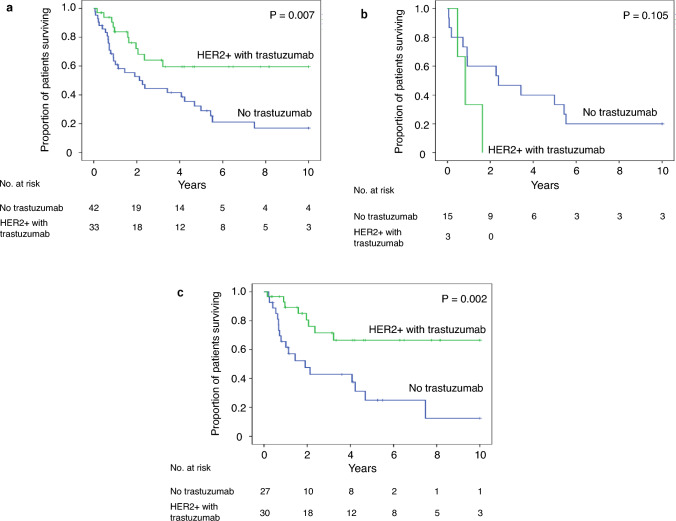
Kaplan–Meier estimates of overall survival in patients BC and ARCM according to trastuzumab therapy. **a** All phase, **b** early phase, and **c** late phase, respectively. Patients with ARCM treated with or without trastuzumab are shown as green and blue lines, respectively. Statistically significant differences were determined using the log-rank test

Survival rates after the first administration of anthracyclines were 71% and 79% at 5 years and 33% and 69% at 10 years in patients with ARCM and non-ARCM, respectively. (log-rank test, *P* < 0.001) (Fig. 4a). Prognostic differences were absent between patients in the early and late phases; however, the survival rate tended to improve in patients in the late phase (*P* = 0.058) (Fig. 4b). The 5-year survival rates in the early and late phases were 28% and 45%, respectively. Patients were divided according to the use of trastuzumab, and the prognostic impact of each phase was analyzed. Trastuzumab users had a better prognosis in the late phase (*P* = 0.002) (Fig. 5c). The 5-year survival rates were 64% and 26% in the trastuzumab and non-trastuzumab groups, respectively.

### Prognostic risk factors in patients with ARCM in multivariate analysis

In all patients, HER2 positivity with trastuzumab therapy, body mass index, metastasis, and NT-proBNP level were positively associated with death according to the crude Cox proportional hazards models (Table 2a). Multivariate analysis revealed that HER2 positivity with trastuzumab therapy, body mass index, and metastasis were independent risk factors for mortality within 10 years. In the late phase, trastuzumab administration and metastases were risk factors for mortality (Table 2b).

**Table 2:** Cox proportional hazards models for factors associated with death within 10 years, (a) Crude hazard ratios for death associated with trastuzumab on HER2 (+) and other prognostic factors related to BC and HF, (b) Adjusted hazard ratios for death associated with trastuzumab on HER2 (+) and possible confounding factors

	Overall			Late phase		
	HR	(95% CI)	*P* value	HR	(95% CI)	*P* value
(a)						
Trastuzumab on HER2 ( +) (yes/no)	0.40	(0.20–0.79)	0.009	0.29	(0.13–0.67)	0.004
Age (per + 1 year)	1.00	(0.97–1.04)	0.884	1.02	(0.97–1.07)	0.516
BMI (per + 1.0 kg/m^2^)	0.90	(0.81–0.99)	0.029	0.87	(0.75–1.00)	0.054
Serum total protein (per + 1 mg/dL)	0.67	(0.42–1.07)	0.093	0.69	(0.36–1.32)	0.261
Metastasis (present = stage 4/absent = stage 3 or less)	7.19	(3.00–17.21)	0.000	7.38	(2.90–18.77)	0.000
Doxorubicin equivalent cumulative dose (per + 100 mg/m^2^)	1.11	(0.98–1.24)	0.098	1.09	(0.94–1.25)	0.255
Estrogen receptor (positive/negative)	1.35	(0.73–2.52)	0.340	1.26	(0.57–2.75)	0.568
Progesterone receptor (positive/negative)	1.34	(0.72–2.49)	0.353	1.09	(0.50–2.37)	0.835
Heart disease (present/absent)	0.64	(0.23–1.80)	0.399	0.77	(0.26–2.22)	0.623
Low LVEF (less than 35%, yes/no)	1.22	(0.56–2.64)	0.619	0.89	(0.33–2.36)	0.808
NT-proBNP (per + 100 pg/mL)	1.01	(1.00–1.03)	0.038	1.01	(1.00–1.03)	0.041
History of HF hospitalization (yes/no)	0.82	(0.43–1.57)	0.546	0.91	(0.40–2.04)	0.811
ACEIs or ARBs	0.77	(0.41–1.44)	0.407	0.80	(0.37–1.73)	0.568
Beta blockers	0.68	(0.34–1.33)	0.254	0.63	(0.28–1.42)	0.266
MRAs	0.69	(0.36–1.31)	0.254	0.63	(0.28–1.42)	0.262
(b)						
Trastuzumab on HER2 ( +) (yes/no)	0.44	(0.21–0.90)	0.024	0.24	(0.10–0.56)	0.001
BMI (per + 1.0 kg/m^2^)	0.90	(0.82–0.99)	0.027	0.93	(0.80–1.07)	0.300
Metastasis (present = stage 4/absent = stage 3 or less)	5.85	(2.43–14.11)	0.000	7.18	(2.75–18.75)	0.000

*ACEIs* angiotensin-converting-enzyme inhibitors, *ARBs* angiotensin receptor blockers, *BC* breast cancer, *BMI* body mass index, *CI* confidence interval, *eGFR* estimated glomerular filtration rate, *HER2* human epidermal growth factor receptor type2, *HF* heart failure, *HR* hazard ratio, *LVEF* left ventricular ejection fraction, *NT-proBNP* N-terminal pro-brain natriuretic peptide

## Discussion

To the best of our knowledge, this represents the longest and largest epidemiological survey on ARCM in an Asian population of women with BC. In this study, we aimed to investigate the changes in ARCM after the therapeutic revolution by trastuzumab therapy in BC. This is because the prognosis of cardiomyopathy is significantly influenced by the prognosis of the underlying disease [23]. Furthermore, improved prognosis has changed not only in epidemiology but also in clinical practice in a community. The major findings of this study are as follows: (1) although the incidence of ARCM has decreased and is currently approximately 2% (Fig. 2), the number of survivors is increasing (Fig. 3). (2) The prognosis of ARCM was worse than that of non-ARCM (Fig. 4a); however, there has been an improvement trend since 2007 (Fig. 4b). (3) Since 2007, the survival rate of patients with ARCM treated with trastuzumab has been significantly better than that of patients with ARCM without trastuzumab. Nevertheless, the survival rate of patients with ARCM who did not receive trastuzumab has remained poor since 1990 (Fig. 5b, c). These findings suggest that the recent rise in HER2-positive ARCM cases treated with trastuzumab, along with their improved prognosis, likely contributes to more patients with BC and ARCM surviving.

The incidence of ARCM has decreased since 1990 (Fig. 2). We speculate that the decreased incidence of ARCM may be due to a decrease in the amount of anthracycline used in individual patients. Throughout the history of advances in chemotherapy, efforts have been made to reduce the use of anthracyclines [9, 10, 13, 14]. Notably, the amount of anthracycline used in the late phase was lower than that used in the early phase of ARCM (Table 1). In the early phase, anthracycline-based systemic treatments, which resulted in a substantial survival benefit but at the cost of cardiotoxicity, were widely adopted in our community. In the late phase, HER2-targeted therapies, including trastuzumab, have gained acceptance, enriching, and enhancing the integrated therapy options available for BC [24]. Consequently, HER2-positive patients become less dependent on anthracyclines in the late phase. The incidence of ARCM in previous reports varied according to the region, patient characteristics, chemotherapy regimens, and definitions of cardiac dysfunction in the research cohort. In a prospective study, Cardinale et al. found that 9.7% of 1344 patients with BC treated with anthracyclines had systolic dysfunction within 1 year [25]. In the Aphinity trial, 0.5% of patients developed symptomatic HF and 2.8% had subclinical left ventricular systolic dysfunction within 1 year [26]. These figures are lower in Japan than in other Western countries. In a claims-based data analysis in Japan, the incidence of HF was 1.0% over 18 months [27]. In a prospective observational study of trastuzumab-treated patients in Japan, encompassing 73% of anthracycline users, the 3-year cumulative incidence of Common Terminology Criteria for Adverse Events grade 3–4 cardiotoxicity was 0.54% [28]. Although exact comparisons cannot be made due to different conditions, in general, our incidence of ARCM in the late phase was higher than that in previous reports in Japan and close to that of the affinity trial [26] (Fig. S1).

Despite the decrease in incidence, the number of surviving patients with ARCM increased (Fig. 3b). We speculated that this increase was caused by an increase in the number of patients treated with anthracycline and improved prognosis. In fact, our number of patients treated with anthracycline increased more than before, and the majority of our patients survived (Fig. 3a). Because ARCM develops in these populations, an increase in the population leads to an increase in ARCM. The prognosis of ARCM patients was worse than that of patients with non-ARCM (Fig. 4). Compared with the National Clinical Database [15], our patients with ARCM had more metastases and more triple-negative histology (Table 1). Because both metastasis and triple-negative histology are poor prognostic factors, patients with ARCM might have a worse prognosis than patients without ARCM in our cohort. However, an improved prognosis was observed in the late phase (Fig. 4b). We observed a favorable prognosis in the ARCM subgroup of ARCM with trastuzumab (Fig. 5c). Trastuzumab therapy was an independent predictor of late-phase survival (Table 2b). A recent prognostic improvement in BC was reported by Caswell-Jin et al. in the US [6]. According to their simulations, the greatest change in survival after metastatic recurrence occurred between 2000 and 2019, from a mean of 1.9 years to a mean of 3.2 years. The median survival for estrogen receptor (ER)-positive/HER2-positive BC improved by 2.5 years, that for ER-negative/HER2-positive BC improved by 1.6 years, and that for ER-negative/HER2-negative BC improved by 0.5 years. Our findings regarding favorable survival in subgroups of patients with ARCM treated with trastuzumab are also consistent with their report. Patients with HER2-positive ARCM likely benefitted from trastuzumab, a more effective targeted therapy than anthracyclines, and the improved prognosis increased the number of survivors in our cohort.

Felker et al. reported that the prognosis of secondary cardiomyopathy varies depending on the underlying disease [23]. In this study, we revealed that prognosis differs depending on the subtype of the underlying disease. HER2 negative BC with metastasis has poor prognosis in ARCM. Most patients had been treated with anthracyclines; the cumulative dose was nearly the upper limit, and few alternative treatments remained. In contrast, HER2-positive BC treated with trastuzumab has a favorable prognosis for ARCM. Notably, nearly half of the patients enrolled in the late phase were HER2-positive and treated with trastuzumab. The cumulative dose of anthracyclines decreased significantly during the late phase. Ewer et al. described cardiac dysfunction caused by anthracycline as Type I CTRCD, and cardiac dysfunction caused by trastuzumab as Type II CTRCD [11]. In other words, our patients with ARCM in the early phase may have mainly had Type I CTRCD, and in the late phase, hybrids of types I and II may have been prevalent. The increase in patients with ARCM in our community during the trastuzumab era confirms previous reports that the addition of trastuzumab to anthracyclines leads to an increased risk of HF but is significantly outweighed by the benefits of improved cancer survival with trastuzumab [24, 29].

Our median time from the last dose of anthracycline to ARCM was 14.3 months (Table 1), which exceeds durations reported previously [25, 26]. The interval depends on echocardiography frequency and follow-up duration. Active surveillance with echocardiography every few months [25, 26] detects cardiac dysfunction earlier than biennial [30] or random surveillance [29]. Long-term studies reveal both early- and late-ARCM, demonstrating a cumulative increase in ARCM over time [7, 25, 26, 29, 30]. Including more cases of late-onset ARCM extends the interval from the last dose of anthracycline to ARCM. For example, a recent US study with a median follow-up of 9.9 years reported a median interval of 6.6 years from BC therapy to cardiac dysfunction [30]. Given methodological variations across studies, caution is necessary for when comparing results.

### Study limitations

Although our study is large, multicenter, and long term, it also has limitations. First, our community lacked a systematic strategy for detecting asymptomatic ARCM. Therefore, echocardiography was performed at the discretion of individual physicians when heart failure was suspected. The average number of echocardiography performed before diagnosing ARCM was low, with 0.7 in the early phase and 2.0 in the late phase. Patients with late-phase ARCM underwent baseline evaluation and one assessment until detecting LVEF < 50%. Although the difference in echocardiography frequency between early and late phases was minimal, more frequent testing might have detected milder cardiac dysfunction and improved survival in the late phase. Second, we could not update the echocardiographic definition of cardiac dysfunction to include modern criteria like global longitudinal strain, as these data were not uniformly available for all patients. Consequently, we relied solely on LVEF. Nevertheless, overall, 77% of our cases met the IC-OS 2021 Consensus CTRCD definition (32 moderate cases and 26 severe cases) [31]. Third, baseline evaluation was not performed in half of the patients in early ARCM. A survey in Niigata City during the early phase indicated that the prevalence of women aged 40–70 years with LVEF < 50% was < 1% [32]. Hence, it is presumed that most women with BC had normal cardiac function before treatment. Fourth, although some guidelines recommend echocardiography within 1 year after completing an anthracycline-containing regimen in the late phase [22], adherence to this recommendation was not evaluated in our community. No existing guidelines in Japan recommended regular echocardiography monitoring throughout the observation period. As most patients appeared to be diagnosed with ARCM based on HF symptoms, there may be a tendency for delayed diagnosis or progression of HF. Consequently, our ARCM cases may not represent mildly symptomatic or asymptomatic ARCM.

## Conclusion

By integrating the BC registries of three cancer centers and observing them over a long period, we could detect the characteristic change of ARCM with BC in our community. The prognosis of ARCM was worse than that of non-ARCM; however, the prognosis of HER2-positive ARCM has improved, and the number of survivors has increased in the trastuzumab era. However, the prognosis for HER2-negative ARCM remained poor. A multidisciplinary team, including oncologists and cardiologists, should understand the importance of this subtype in the treatment of patients with BC and ARCM and support each patient.

## Supplementary Information

Below is the link to the electronic supplementary material.

Fig. S1 Cumulative incidence rate curves for ARCM from the start of anthracycline administration. The curve of incidence from 1990 to 2020 is shown in (a), and the curves of incidence divided into 1990–2006 (early phase) and 2007–2020 (late phase) are shown in (b). Statistically significant differences were determined using the log-rank test

Fig. S2 Scattergrams and regression lines between the cumulative number of patients with BC or ARCM in the early or late phases. The regression equations are indicated for each of the four groups. a. Patients treated with anthracycline. b. Patients diagnosed with ARCM

Supplementary file3 (PDF 191 KB)

## Data Availability

The datasets generated and/or analyzed during the current study are available from the corresponding author upon reasonable request.
