# Supplementary figures and images for: Increasing survivors of anthracycline-related cardiomyopathy with breast cancer in trastuzumab era: thirty-one-year trends in a Japanese Community
Source: Breast Cancer. 2024 Aug 13;31(6):1080–91. doi: 10.1007/s12282-024-01623-0 (PMC11489246; doi:10.1007/s12282-024-01623-0)

Fig. S1

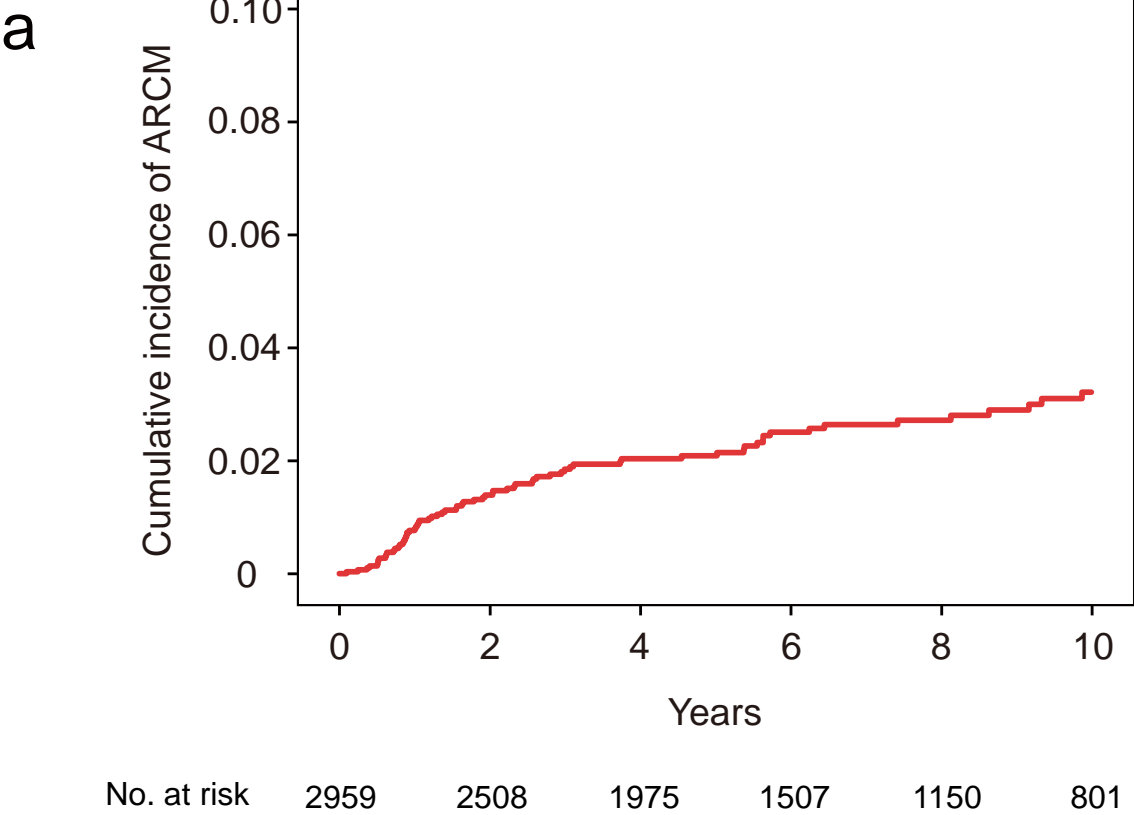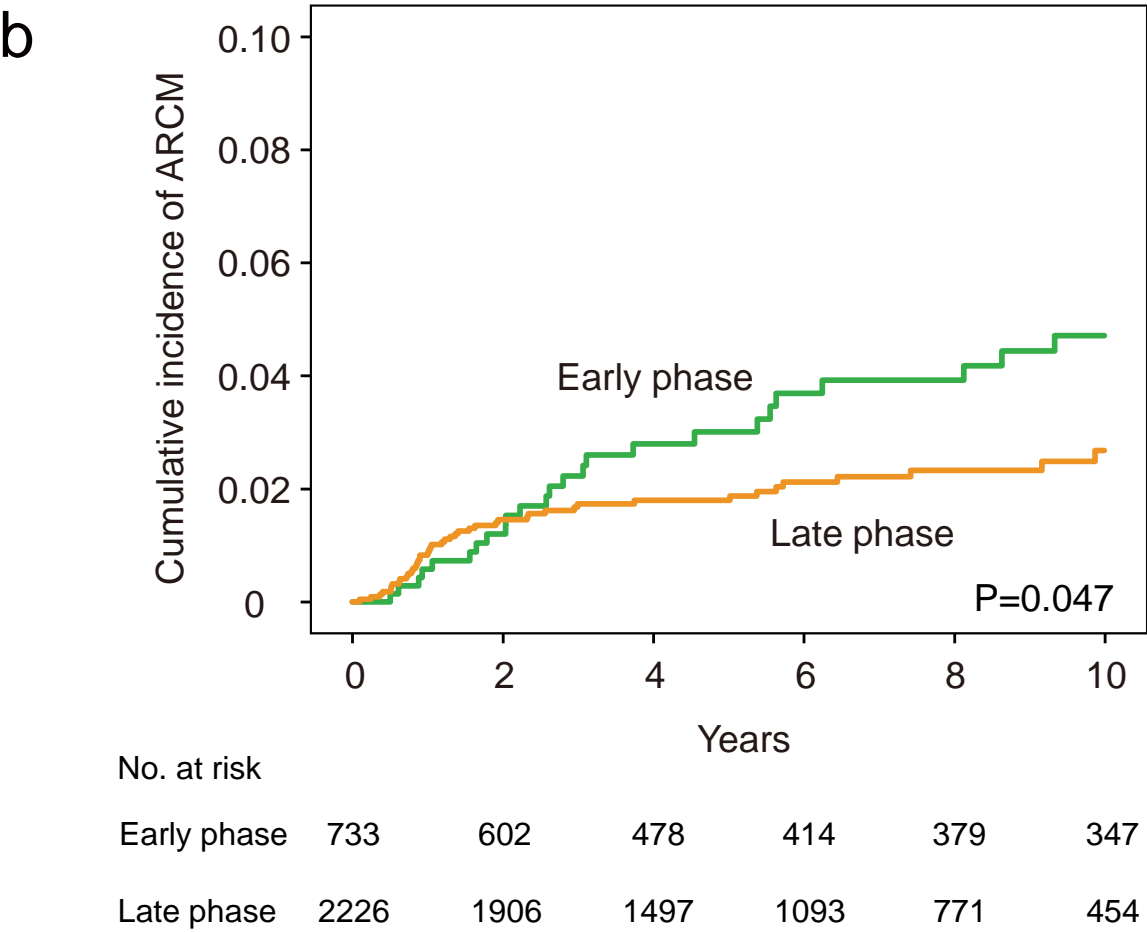

Supplement: Supplementary file 1 — Fig. S1 Cumulative incidence rate curves for ARCM from the start of anthracycline administration. The curve of incidence from 1990 to 2020 is shown in (a), and the curves of incidence divided into 1990–2006 (early phase) and 2007–2020 (late phase) are shown in (b). Statistically significant differences were determined using the log-rank test [file 12282_2024_1623_MOESM1_ESM.pdf]

Fig. S2

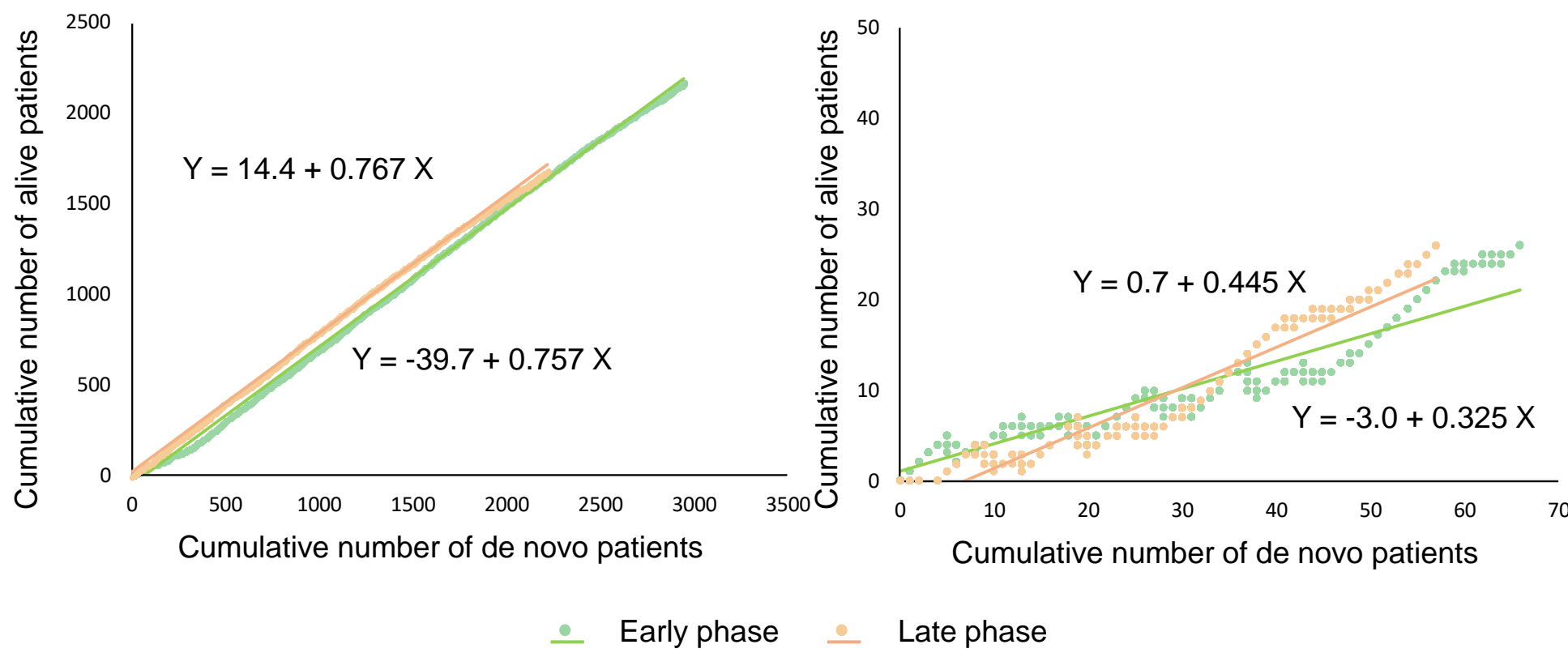

a. Patients treated with anthracycline

b. Patients with ARCM

Supplement: Supplementary file 2 — Fig. S2 Scattergrams and regression lines between the cumulative number of patients with BC or ARCM in the early or late phases. The regression equations are indicated for each of the four groups. a. Patients treated with anthracycline. b. Patients diagnosed with ARCM [file 12282_2024_1623_MOESM2_ESM.pdf]
