# Supplementary material for: Increasing survivors of anthracycline-related cardiomyopathy with breast cancer in trastuzumab era: thirty-one-year trends in a Japanese Community
Source: Breast Cancer. 2024 Aug 13;31(6):1080–91. doi: 10.1007/s12282-024-01623-0 (PMC11489246; doi:10.1007/s12282-024-01623-0)
Supplement: Supplementary file 3 — Supplementary file3 (PDF 191 KB) [file 12282_2024_1623_MOESM3_ESM.pdf]

Table S1

Regression coefficients that predict the number of patients alive based on the cumulative number of de novo patients, calendar year phase, and ARCM status (Fig. 3).

X = cumulative number of patients treated with anthracyclines

Y = cumulative number of alive patients

Y = a+bX

Early phase, patients treated with anthracyclines

Y = -39.7 + 0.757 X

Late phase, patients treated with anthracyclines

Y = -39.7 + 54.1 + (0.757 + 0.010*) X = 14.4 +767X

Early phase, ARCM

Y = -39.7 + 40.4 + (0.757 - 0.432) X = 0.7 + 0.325X

Late phase, ARCM

Y = -39.7 + 40.4 + 54.1 - 57.8 + (0.757 - 0.432 + 0.010* + 0.110†) X = -3.0 + 0.445X

	Coefficient	(95% CI)		*P*
De novo (n)	0.757	(0.757	, 0.757)	<0.001
Calendar phase (late / early)	54.1	(53.1	, 55.1)	<0.001
ARCM (yes / any)	40.4	(39.8	, 40.9)	<0.001
De novo × Calendar phase	0.010	(0.009	, 0.010)	<0.001
De novo × ARCM	-0.432	(-0.446	, -0.418)	<0.001
Calendar phase × ARCM	-57.8	(-59.2	, -56.5)	<0.001
De novo × Calendar phase× ARCM	0.110	(0.08	, 0.14)	<0.001
Intercept	-39.7	(-40.09	, -39.26)	<0.001

*†: Interaction term of the regression coefficient (b) for the increase in the number of surviving patients (Y) from the increase in the number of treated patients (X) with the calendar phase, P<0.001.

In the anthracycline-treated patient group, the regression coefficients were significantly, but only slightly, elevated in the late phase than in the early phase (+0.010*). However, the increase in the coefficients was much greater in the ARCM group than in the anthracycline-treated patient group (+0.110†).
